# Effect of aquatic exercise versus standard care on paraspinal and gluteal muscles morphology in individuals with chronic low back pain: a randomized controlled trial protocol

**DOI:** 10.1186/s12891-023-07034-0

**Published:** 2023-12-18

**Authors:** Brent Rosenstein, Chanelle Montpetit, Nicolas Vaillancourt, Geoffrey Dover, Najmeh Khalini-Mahani, Christina Weiss, Lee Ann Papula, Antonys Melek, Maryse Fortin

**Affiliations:** 1 Department of Health, Kinesiology and Applied Physiology, Concordia University, 7141 Sherbrooke Street W, Montreal, QC Canada; 2 School of Health, Concordia University, Montreal, QC Canada; 3 McGill Centre for Integrative Neuroscience, Montreal Neurological Institute, Montreal, QC Canada; 4 CRIR – Centre de réadaptation Constance-Lethbridge du CIUSSS COMTL, Montreal, QC Canada

**Keywords:** Non-specific low back pain, Aquatic therapy, Paraspinal muscle, Gluteal muscle, MRI, Function, Quality of life

## Abstract

**Background:**

Low back pain (LBP) is one of the most disabling diseases and a major health issue. Despite the evidence of a link between paraspinal and gluteal muscle dysfunction and LBP, it is unknown whether aquatic exercises can lead to improvements in paraspinal and gluteal muscle morphology and function, and whether improvements in overall muscle health are associated with improvements in patients’ outcomes. The unique properties of water allow a water-based exercise program to be tailored to the needs of those suffering from LBP. This study uses magnetic resonance imaging (MRI) to investigate the effect of an aquatic exercise program versus standard exercise on 1) paraspinal and gluteal muscle size, quality and strength and 2) pain, disability, and psychological factors (pain related fear, depression, anxiety, sleep quality) in chronic LBP.

**Methods:**

This study will include 34 participants with chronic non-specific LBP and moderate to severe disability, aged between 18 and 65, who will be randomly assigned (1:1) to the aquatic exercise group or land-based standard care exercise group. Both groups will receive 20 supervised sessions, twice per week over 10 weeks. MRIs will be obtained along the lumbosacral spine (L1-L5) and pelvis at the start and end of the intervention to assess the effect of each exercise intervention on paraspinal and gluteal muscle size and quality. Pre- to post-intervention changes in all outcomes between each group will be assessed, and the association between the changes in back muscle quality and clinical outcomes will be examined. Between-subjects repeated measure analysis of variance will be used to examine the changes in paraspinal muscle morphology over the different time points. Linear mixed models will be used to assess whether baseline scores can modify the response to the exercise therapy treatment.

**Discussion:**

This study will determine if water-based exercises targeting the lower back and gluteal muscles can lead to important changes in muscle quality and function, and their possible relation with patients’ pain and functional improvements. Our findings will have strong clinical implications and provide preliminary data to design a community program to better support individuals with chronic LBP.

**Trial registration:**

NCT05823857, registered prospectively on April 27^th^, 2023.

**Supplementary Information:**

The online version contains supplementary material available at 10.1186/s12891-023-07034-0.

## Background

Low back pain (LBP) is a well-recognized and significant public health concern [1, 2]. The healthcare-related cost for chronic LBP and social consequences are substantial for the society [2, 3]. In Canada, LBP related medical costs range between 6 and 12 billion dollars each year, and continue to increase [4]. In addition, impairments in strength [5, 6], flexibility [7, 8], endurance [9], and obesity [10] are well documented in individuals with chronic LBP. Exercise therapy is currently the most widely used form of conservative treatment for chronic LBP, with recent reviews supporting its effectiveness [11, 12]. Therefore, exercise therapy is recommended as a first-line treatment for people with chronic LBP [13], especially for the improvement of pain, quality of life, depression and disability/functional status [13–21]. Given the body of evidence linking paraspinal muscle morphological changes (e.g. atrophy, fatty infiltration, asymmetry), gluteal functional changes (e.g., decreases in strength and muscle activation) [22–24] and LBP [25, 26], and lack of spinal stability due to impairments in the trunk and paraspinal muscles [27], many exercise interventions focus on activating of these muscles [17–19].

However, it is difficult for those with LBP to perform strengthening exercises without avoiding the weight load on their spine [28]. In addition, fear avoidance beliefs which refer to fear of physical movement and work activities that may elicit pain is very common in individuals with chronic LBP [11, 12]. One form of exercise that has been suggested as a promising and safer treatment alternative for individuals with LBP is aquatic exercise, with less risk of injury and difficulty performing exercises [14, 29–35]. Water immersion decreases axial loading of the spine and, through the effects of buoyancy, allows the execution of movements that are usually difficult to perform on land by reducing stress in joints [36, 37]. The unique properties of water, such as buoyancy, allow a water-based exercise program to be tailored to the needs of those suffering from LBP. Aquatic exercise may improve pain and disability [38], and maintain quality of life in individuals with chronic LBP [35], especially in individuals with low levels of physical fitness [39, 40]. Improvements in muscle strength [41] and cardiovascular fitness [31, 42] through continuous limb movements against water resistance have been reported as well. A recent systematic review and meta-analysis on the effect of aquatic therapy in LBP, showed that this may not only be an effective alternative to conventional LBP treatments, but also a safe and enjoyable substitute to improve physical function [38]. These findings suggest the potential benefits of aquatic exercise for individuals with chronic LBP.

Recent reviews concluded there is no clear particular form of exercise that is more effective than others [17–19]. Interestingly, when comparing aquatic therapy to land-based exercise in individuals with chronic LBP, some study results have showed no significant differences in decreased pain levels or improved functional ability between the two exercise types [31, 34]. However, one study showed better improvements in functional ability in the aquatic therapy group compared to the land-based exercise group [35]. Moreover, Psych et al. showed that aquatic therapy produced comparable back and gluteal muscle activation and intensity to land-based exercise [43]. Overall, there are some inconsistencies between studies comparing aquatic therapy to land-based exercise in individuals with chronic LBP.

While many exercise intervention studies reported improvements in patient-reported outcomes such as pain, physical function, and depression, few comprehensive imaging studies have examined these outcomes in relation to muscle morphology (e.g. hypertrophy, fatty infiltration) and functional improvements (e.g. strength, endurance, contraction), and many have been critiqued for their low quality and high potential of methodological bias [44]. Furthermore, we are not aware of any imaging studies that have specifically assessed the effect of aquatic therapy on paraspinal and gluteal muscles morphology and function. Lastly, while mobile health applications to manage LBP have been used in the past, it remains unclear if they are feasible to use both in a standard clinical setting and during a specific LBP exercise intervention. Most previous studies have used such applications as a stand-alone intervention. Interestingly, a randomized controlled trial (RCT) investigated the efficacy of a stand-alone mobile-Web intervention to help users adopt self-management strategies for non-specific LBP [45]. This tool was effective in the prevention and management of LBP occurrences, on current back pain, behavioral, and worksite outcomes, by tailoring content to users’ preferences and interests. Another study showed that an iPad application screening for pain contributors and providing personalized education for older adults with chronic LBP, had highly rated usability and utility [46]. These studies support the potential value a technological intervention has in this population, and could possibly be applied in clinical settings to educate patients about LBP, to facilitate patient-provider communication for personalized treatment strategies, and enhance compliance. The application “play the pain” was specifically developed for subjects dealing with chronic pain, however, it has not been used or tested in a clinical population (e.g., chronic LBP) to date. Therefore, the purpose of this study is to investigate the effect of an aquatic therapy exercise program versus standard care on 1) paraspinal and gluteal muscle size, composition (e.g. fatty infiltration) and strength, 2) pain, disability, quality of life, and psychosocial factors (e.g. kinesiophobia, catastrophizing, anxiety, and depression) in individuals with chronic LBP, and 3) to evaluate the feasibility, adherence and satisfaction of participants to use the “play the pain” app to track their pain levels and the activities they used to cope with the pain.

### Hypotheses

It is hypothesized that participants in the aquatic therapy group will show a significant improvement in 1a) paraspinal and gluteal muscle size and 1b) paraspinal and gluteal strength following the intervention, as compared to the standard care group. We also hypothesized that both groups will show improvements in pain, disability, quality of life, and psychosocial factors following the completion of the intervention, however the effects will be greater in the aquatic group as compared to the standard care group. Finally, we hypothesized that it will be feasible to implement the app in both the standard care and aquatic exercise groups, and that participants will be compliant and satisfied with the application.

## Methods/design

### Study design

This will be a two-arm prospective RCT. This protocol was reported in accordance with the SPIRIT guidelines [47] and CERT recommendation for Exercise Interventional Trials [48].

### Study setting

This study will be conducted at the Concordia University School of Health, Montreal, Canada (NCT05823857). The proposed project was approved by the Central Ethics Research Committee of the Quebec Minister of Health and Social Services (# CCER-21–22-35). All participants will sign a consent form prior to beginning the study.

### Participant recruitment

Study participants will be recruited from the School of Health Athletic Therapy clinic in Montreal, Canada and through poster and media advertising (e.g. email blast by the School of Health) as this has been shown to be an effective manner to attract participants [49]. If individuals’ express interest in participating in the trial, a member of the research team will contact them to discuss the study further, interview them, confirm eligibility, obtain consent, and enroll participants.

### Participants

#### Inclusion criteria

Individuals will be eligible to participate in the trial if they meet the following criteria:

- Chronic non-specific LBP (> 3 months), defined as pain in the region between the lower ribs and gluteal folds, with or without leg pain.
- Currently seeking care for LBP.
- Aged between 18 to 65 years old.
- English or French speakers
- Have a score of “moderate” or “severe” disability on the modified Oswestry Low Back Pain Questionnaire
- Do not currently engage in sports or fitness training specifically for the lower back muscles (3 months prior the beginning of the trial).

#### Exclusion criteria

Participants will be excluded if they meet one of the following criteria:

- Evidence of nerve root compression or reflex motor signs deficits (e.g., weakness, reflex changes, or sensory loss with same spinal nerve),
- Previous spinal surgery or vertebral fractures,
- Other major lumbar spine structural abnormalities (e.g., spondylolysis, spondylolisthesis, or lumbar scoliosis > 10°)
- Comorbid health conditions that would prevent active participation in exercise programs (e.g. screened with Physical Activity Readiness Questionnaire)

### Randomization

After providing written informed consent, participants will be randomly assigned to treatment groups (1:1) using consecutively numbered sealed opaque envelopes (e.g., computer-generated randomization sequence with permuted blocks) created by an individual not involved in the study.

### Blinding

Since blinding of therapists and participants is generally not possible in exercise intervention trials [50], only the assessor will be blinded.

### Procedure

Eligible participants will be randomized into an aquatic therapy group or land-based standard care group. The intervention period will last 10 weeks, with a frequency of 2 times a week for each group at the School of Health, Concordia University. This frequency of training is in accordance with previous related exercise intervention studies for chronic LBP [19]. The duration of 10 weeks was chosen as strength improvements from training largely occur within that time period [51]. All methods will be carried out in accordance with relevant institutional guidelines and regulations. Written consent form will be obtained from all participants prior any data collection, and will be obtained to publish this paper.

### Swim ex water-based trunk stabilization group (Swim Ex)

Participants in this group will perform trunk stabilization, which will be based on a variety of aquatic exercises in different positions, intended to activate the multifidus and transverse abdominis in a co-contraction. Additionally, hip and gluteal specific exercises will be completed to promote strengthening of the gluteus maximus, gluteus medius, and gluteus minimus muscles. The aim of these exercises is to enhance the strength and dynamic stability of the spine and its surrounding musculature in a functional and yet non-weight-bearing way. Each exercise will be performed 10 times while sustaining the muscles co-contraction for 5–10 s and will be progressed gradually by adding low load to the muscles by means of external leverage at the limbs (discs, hand paddles), resistance material (kickboard, dumbbell floats), water current and/or leg length, and by gradually adding repetitions. Each training session (~ 60 min) will be privately supervised one-on-one by a certified Athletic Therapist (AT) and take place in the School of Health AT Clinic/Swim Ex pool. The level of difficulty will be increased using the Borg Rating of Perceived Exertion Scale (1–10 scale) (e.g. set level three = moderate or four = somewhat hard) and the Category Ratio scale (0–10 pain scale) [52]. This aquatic exercise program (refer to Table 1) was adapted from a recent study in adults with chronic LBP [43].

**Table 1:** Aquatic exercise program group

	**Aquatic Exercises**	**Volume**	
**Warm-Up** (10 min)	**Water walking:** forwards, backwards, sideways—left and right. Progressively increase walking speed	3 min (30 s / direction)	
	**Small hops:** feet shoulder width apart, squat, then jump and bring feet close together and squat back down	1 min (30 s/ direction)	
	**Leg kicks:** kick front, kick out by side, kick back	1 min (30 s / leg)	
	**Marching:** raise knees, heel to buttocks—as tolerated	2 min (1 min each)	
	**Horizontal kicking:** on stomach, while holding onto edge of pool, kick legs and try to maintain body horizontal. Use floating device under the waist to modify difficutly	1 min (30 s each)	
	**Upper body oscillations:** Moving both arms in an oscillatory fashion from in front of you to out by your side	1 min	
	**Bow and Arrow:** rotate the torso by bringing your arms close to your body as if you’re shooting a bow and arrow. Alternate sides	1 min	
**Training** **Session** (​​40 min)	**Multi-directional Lunges:** forward, backward, sideways lunges back and forth	2 sets × 10 repetitions / direction	
	**Squats:** Place both arms by your side. Bend down in a squat position and then return to starting position. Can control speed of exercise done to make it harder	2 sets × 1 – 2 min	
	**Step-Ups:** Using an underwater step, place one foot on the step with hands on your waist and transfer weight to step up	2 sets × 8—12 repetitions / side	
	**Single leg squat:** Stand on one leg with both arms by your side. Perform the single leg squat until the knee moves in front of the toes. Remove hand support to increase difficulty	2 sets × 8 – 12 repetitions / side	
	**Standing Hip Abduction:** Stand on one leg, while either maintaining your balance or holding on to the side of the pool. Bring the leg that you aren’t standing on outwards as far as possible as long as the movement comes your hip. Use an elastic band above the ankles to make the exercise harder	2 sets × 10 repetitions / side	
	**Standing Hip Extension:** Stand on one leg, while either maintaining your balance or holding on to the side of the pool. Bring the leg that you aren’t standing on behind you as far as possible as long as the movement is only coming from your hip. Use an elastic band above the ankles to make the exercise harder	2 sets × 10 repetitions / side	
	**Concentric chest press + row:** Lunge or squat down to chest-deep water and hold dumbbell floats vertically between hands with arms fully outstretched in front just below the water surface. Pull dumbbell floats close to the chest (bringing arms backwards towards body) and then push forwards to starting position, while staying put	2 sets × 1—3 min	
	**Underwater punches:** Lunge down to have your arms just below the water surface, with one arm fully outstretched in front and the other close to body. Holding Aqualogix dumbbells, perform alternate punching with each arm	2 sets × 1 – 3 min repetitions	
	**Concentric Shoulder Flexion + Shoulder Extension:** Place one arm by side and the other outstretched in front just below water surface with palms facing direction of movement. Alternately bring one arm upwards just below the water surface while bringing the other arm to the side. Use hand paddles to increase resistance and change the front leg between sets	2 sets × 10—20 repetitions	
	**Knee Raises:** Hold the aquatic dumbbell in each hand with arms by sides while in a seated position. Raise one knee until thigh is parallel to the surface of the water. Alternate legs	2 × 10—20 repetitions	
	**Horizontal Woodchopper:** Have your hands with your arms fully outstretched in front just below water surface. Rotate your torso gradually as far to one side as possible and then back to the midline. Alternately do the same with other side	2 sets × 10—20 repetitions / side	
	**Diagonal Woodchopper:** Have your hands with your arms fully outstretched in front just below water surface. Rotate your torso gradually as far to one side as possible while bringing the hands down diagonally and then back to the midline. Alternately do the same with other side	2 sets × 10 – 20 repetitions / side	
	**Water Ab Rollout:** Start in upright posture with arms outstretched in front of you and your hands resting on the surface holding dumbbell floats. Slowly move the dumbbell floats forwards while keeping your body in a neutral posture tilting on the tips of the toes, and then return to starting position. You should feel the movement working your abdominal muscles	2 sets × 10—15 repetitions	
**Cool-Down** (10 min)	**Free water-activity:** walking, standing, swimming	1 min	
	**Stretching / Mobility exercises:** Hip CARs (controlled articular rotations)	2—5 × each leg	
	**Stretching/Mobility exercises:** Standing quad stretch	2 × 30 s per muscle group	
	**Stretching/Mobility exercises:** Standing hamstring stretch (extend leg + lean in)	2 × 30 s per muscle group	
	**Stretching/Mobility exercises:** Anterior chain stretch: grab arms behind back + open up chest	2 × 30 s per muscle group	
	**Stretching/Mobility exercises:** Seated Fig. 4 stretch	2 × 30 s per muscle group	
	**Stretching/Mobility exercises:** Standing open book (hold onto side of pool, lift leg + rotate away)	2 × 30 s per muscle group	
	**Relaxation:** Lay on your back with a noodle supporting you under your waist and occiput and take deep breaths	1 – 5 min	

Exercise will progress over the weeks by adding resistance to certain exercises (water current, ankle weights, hand paddles, dumbbell floats, kickboards, resistance bands, discs), as well as increasing in the number of repetitions

### Standard care group

Participants in this group will receive the standard LBP treatment in the School of Health Athletic Therapy clinic and will be privately supervised one-on-one by a certified AT who will be conducting the sessions. The AT will complete a thorough assessment of the eligible participants with chronic LBP and administer a range of interventions including stretching, strengthening and stabilization exercise, aerobic conditioning, and manual mobilization techniques. No attempt will be made to try to regulate/standardize the treatment received, however information about pain medication and co-interventions will be collected throughout the duration of the study.

### Data collection

All outcomes will be obtained at baseline for both intervention groups. All baseline assessments (e.g. MRI, strength, questionnaires) will be repeated at the end of the intervention during a 10-week follow-up (refer to Table S1 in Appendix). All self-reported questionnaires will be completed in-person using paper forms. Imaging outcomes (e.g., MRI) and lumbar extensor muscle assessments (e.g., strength) will be acquired at the School of Health, Concordia University. Demographic characteristics will be acquired via a self-reported questionnaire at baseline, after randomization.

### Outcome measures

#### Primary outcome measure

Multifidus muscle size and composition (e.g., fatty infiltration).

##### MRI assessment of multifidus muscle morphology

All participants will undergo baseline routine lumbosacral MRI evaluation prior the beginning of the exercise intervention using the School of Health’s 3-T General Electric (Chicago, IL, USA) MRI machine. Axial T2-weighted and IDEAL (Lava-flex, 2 echo sequence) will be obtained from L1 to L5 to assess the multifidus morphology and composition. Bilateral manual segmentation of regions of interest (ROI) representing the cross-sectional area (CSA) of the multifidus muscle will be acquired on axial T2-weighted slices from the most cranial aspect of the first lumbar vertebra (L1) to the most caudal aspect of the fifth vertebra (L5) to calculate the summative 3D volume of the right and left side at each level (more accurate assessment than single slice). IDEAL axial water and fat images will be used to calculate the percent fat-signal fraction: %FSF = (Signal_fat_/[Signal_water_ + Signal_Fat_] × 100) of the muscle. The Horos DICOM viewer software will be used for imaging analysis.

#### Secondary outcome measures

1) erector spinae muscle size and composition 2) gluteal muscles size and composition 3) lumbar extensor and gluteal muscles strength, 4) pain, 5) disability, 6) health related quality of life, 7) kinesiophobia, 8) catastrophizing, 9) depression/anxiety, 10) level of physical activity and 11) sleep quality.

##### MRI assessment of erector spinae and gluteal muscles morphology

The same method, as described above for the multifidus muscle, will be used to assess the erector spinae morphology and composition at each lumbar level. Similarly, a routine pelvic MRI T2-weighted and IDEAL (Lava-flex, 2 echo sequence) will also be acquired to assess gluteus maximus, gluteus medius and gluteus minimus muscle size and composition. IDEAL axial water and fat images will be used to calculate the percent fat-signal fraction: %FSF = (Signal_fat_/[Signal_water_ + Signal_Fat_] × 100) of each muscle. The Horos DICOM viewer software will be used for imaging analysis.

##### Lumbar extensor muscles strength

Lumbar extensor muscle strength will be evaluated by the MedX lumbar extension machine. Participants’ hips, knees, and pelvis will be secured to the machine to ensure isolation of the lumbar extensor muscles with a fixed axis of movement between the L5-S1 vertebral levels. This dynamometer measures isometric lumbar extension muscular strength (torque) in a seated position and allows dynamic resistance through a full 72° range of motion (ROM). Therefore, the maximum lumbar extension torque will be measured as maximum voluntary isometric contraction (MVIC) in lumbar extensor muscle strength in seven positions: 72°, 60°, 48°, 36°, 24°, 12° and 0° of flexion [53, 54]. Participants will be seated and positioned in the apparatus. A belt will be fastened over the thighs and tightened until they can no longer lift their buttock’s off the seat. A fixation will be positioned a few inches above the participants’ patella, and they will rest their feet on a platform that will also be tightened to restrict any movement of the heels beyond one inch. Participants’ will also have a neck support at the base of their occipital bone. Initial testing will be performed to verify any limitations in their ROM and adjustment for the counterweight [55]. Participants will initially perform a slow controlled warm up for approximately 1 min, and then the maximum strength test will begin [53]. Verbal encouragement will be provided to motivate participants to generate maximum torque. The movement arm of the MedX machine is linked to a load cell that is interfaced with a computer, which will record and calculate torque measurements.

##### Gluteal muscle strength

A hand-held dynamometer (micro FET2; Hoggan Health Industries) will be used to assess gluteal muscle strength. Participants will be placed on a therapy table in a prone position with their arms by their side to assess gluteus maximum muscle strength with the knee at 90 degrees of flexion and the thigh/hip slightly extended (e.g., off the table). Participants will be instructed to maintain this position for 5 s, creating an isometric contraction in the form of a “make” test and asked to exert a maximal force against the hand-held dynamometer. Measurements will be recorded in newton/torque. All participants will have a submaximal practice trial and then 3 measurements will be obtained on each side, with a 30 to 45 s break in between each trial. The mean of the 3 measurements will be used in the analysis. Similarly, patients will be placed in a side lying position with their arms by their side and with their top leg abducted, slightly extended and externally rotated to test the gluteus medius muscle. Again, participants will be instructed to maintain this position for 5 s, while exerting a maximal force against the hand-held dynamometer. Three measurements will be acquired on each side. Two certified ATs will be acquiring the measurements. Hand-held dynamometry has been shown to be a valid [56] and highly reliable tool to assess gluteus muscle strength [57] and has been recommended as a practical standard for clinical setting.

#### Questionnaires

Secondary self-reported outcome measures will include, disability/function status, pain, pain related fear including catastrophizing and kinesiophobia, function, depression, anxiety, level of physical activity and sleep quality obtained from self-reported questionnaires. Participants will be asked to complete the Canadian Minimum Data set, the Short-Form 12 Item Survey questionnaire (SF-12), modified Oswestry Low Back Pain Disability Index (ODI) and numerical pain rating scale will be used to measure patients’ disability/functional status and related pain. The Pain Catastrophizing Scale (PCS) and the Tampa Scale of Kinesiophobia (TSK) will be used to measure pain related fear. The Hospital Anxiety and Depression Scale (HADS) will be used to assess depression and anxiety, and the International Physical activity questionnaire (IPAQ) and Insomnia Severity Index (ISI) will be used to assess the level of physical activity and sleep disturbances, respectively. All questionnaires have previously demonstrated a good level of test–retest reliability and have been validated in patients with chronic LBP [58–67].

##### Disability

The ODI will be used to assess participants’ level of self-reported disability in relation to LBP. It is a 10-item scale in which each item is rated from 0 to 5, where 0 signifies that their pain does not influence that situation and a score of 5 signifies severe disability. Pain, walking, lifting, sitting, standing, personal care, sleeping, travel, sex life, and social life are categories included in the questionnaire. Scores are classified as minimal, moderate, severe, crippled, or bed bound. The ODI has shown good reliability and validity, and therefore is deemed to be the gold standard of measuring disability related to LBP [68].

##### Health related quality of life

The 12-item SF-12 is the condensed form of the past 36-item survey and will be used to measure participants’ health-related quality of life. The 12-item survey consists of eight domains that measure both physical and mental components of health: 1) limitations in physical activities due to health problems, 2) limitations in social activities due to physical or emotional problems, 3) limitations in usual role activities due to physical health problems, 4) bodily pain, 5) general mental health (psychological distress and well-being), 6) limitations in usual role activities due to emotional problems, 7) vitality (energy and fatigue) and 8) general health perceptions. Scores from each of the 12 questions are summed to calculate an overall score between 0 and 100, with a score of 100 representing the highest level of health. Since this is a condensed version of a longer and established questionnaire, it has been widely tested and considered to be both reliable and valid [69, 70].

##### Pain

The Numerical Pain Rating scale (NPR) will be used to measure participants’ level of pain. This tool is a self-reported rating system for pain intensity. The NPR has excellent reliability and validity, and can be used to identify statistical and clinically significant changes in perceived pain [61, 71].

##### Catastrophizing

The PCS is a 13-item questionnaire that will be used to measure participants’ level of catastrophizing. Each item is rated from 0 to 4 for a possible total of 52. The questionnaire focuses on three domains that have been used to portray catastrophizing: attentional focus on pain related thoughts (rumination), exaggeration of painful stimuli (magnification), and adopting a hopeless orientation with coping (helplessness). A higher score indicates a higher level of catastrophizing, with scores above 30 being clinically significant. This measure is both reliable and valid [62, 72].

##### Kinesiophobia

The TSK will be used to assess participants’ fear of movement or reinjury in the presence of pain. The TSK- 11 includes 11 phrases related to kinesiophobia, such as “I’m afraid I might injure myself if I exercise”, with each phrase rated with a Likert scale from 1 to 4. Scores range between 11 to 44 with higher scores indicating increased levels of kinesiophobia. This measure has been found to have high reliability and validity [63].

##### Depression

The HADS is a 14-item questionnaire that will be used to measure participants’ level of depression and anxiety. Seven items are associated with depression and 7 are associated with anxiety. Cognitive, behavioural, and emotional symptoms are involved in the questionnaire. Each item is rated from 0 to 3 with either depression or anxiety having scores between 0 and 21, with 21 as the highest possible level. Scores between 0 to 7, 8 to 10, and 11 to 21 are categorized as normal, borderline, and abnormal/elevated for any domain, respectively. This tool was deemed to be both reliable and valid [64].

##### Sleep

The ISI will be used to measure self-reported quality of sleep. It includes 7 questions that assess the ability to fall asleep, the ability to stay asleep, and sleep effects on daily life. Each question is rated with a Likert-scale from 0 to 4, with lower ratings representing a higher quality of sleep. Scores between: 0 and 7 indicate no clinically significant insomnia, 8 to 14 indicate subthreshold insomnia, 15 to 21 indicate moderate insomnia, and 22 to 28 indicate severe insomnia. Fourteen is commonly used as the cut-off score to detect primary insomnia. This tool has been found to be both reliable and valid [67].

##### Physical activity: international physical activity questionnaire (IPAQ)

The IPAQ will be used to measure participants’ level of physical activity. The IPAQ is a self-reported log of physical activity (METs based on intensity) in minutes per week over a period of 7 days. The level of physical activity is ranked either vigorous (8 MET), moderate (4 MET), walking (3.3 MET) and sitting/rest (1 MET) and must be allocated to the right category. The number of minutes per category is then combined, and the results are then organized as high, moderate, or low physical activity based on the total MET minutes. The reliability and validity of this measure has been demonstrated [66].

#### Exploratory outcomes

1) feasibility of using the application in both groups, 2) adherence of using the application, and 3) participants’ satisfaction with the application.

##### Digital app “play the pain”

Participants in both groups will also be asked to use the digital application “play the pain”. To avoid an inadvertent co-intervention, the “Play’’ function of the app will not be used in the trial. The function of interest for this study is the “track’’ function. Participants will be able to track their pain, their activities, their sleep, their medication use and their emotions. The pain tracking function allows users to describe the location of the pain with an interactive model of the human body. After determining the location of the pain, users are prompted to scale the intensity with a movable cursor from slight pain to extreme pain. The emotion tracking features an interactive web with a wide range of feelings including: anxious, afraid, worried, happy, calm, etc. Each emotion can be clicked on and given an intensity ranging from “a little” to “a lot”. The sleep tracking function tracks the duration of sleep with a dichotomous scale. The two options are “more than 7 h’’ and “less than 7 h’’. After choosing an option, users are asked to describe the quality with a 5-point Likert scale. The options are: “Great”, “Good”, “OK”, “Not good” and “Very bad”. Each option is accompanied with a smiley face visually representing the choice. The medication tracking feature asks if the medication taken is prescribed or not, with an empty box for a description of the type of medication and the dosage. The activity tracking feature has 24 options to choose from. This feature includes physical activity including: exercise, dancing, walking, yoga, swimming, jogging and cycling. Additionally, included are treatment alternatives often used for chronic pain, such as, physiotherapy, cognitive behavioral therapy, meditation, acupuncture and recreational drug use. The last category of the activity tracking feature is focused on hobbies including: gaming, reading, painting and watching television. In addition, there is an “Other” section in order for other activities that were not included in the list to be described. Participants in the study will be asked to use the app on a daily basis. There are no specific guidelines on what should be tracked each day. The philosophy of Play the Pain is to let the user share what is on their mind, and to give the clinician a better understanding of their condition and how patients cope with pain [73, 74]. Participants will be introduced to the application when they come to the School of Health for their first visit, and will be provided with clear instructions on how to install it and use it. Each participant’s account will be linked to an administrative account managed by the research team, allowing us to track each participant and their use of the application. At the end of the intervention, the participants will be asked to complete and exit survey regarding their satisfaction with the use of the application.

## Data monitoring

### Adverse events

Adverse events (e.g., short-term increase in LBP and muscle soreness) will be monitored by the ATs during and following the interventions by asking open ended questions.

### Adherence

Adherence to the exercise intervention will be recorded by the ATs using each participants’ treatment file.

### Co-interventions

At the end of the study, participants will be asked to report any co-interventions (e.g., chiropractic, physiotherapy, osteopathy, massage therapy) during the intervention. Pain medication will be allowed since it is unethical to deny medication, and will be recorded for all participants.

### Data integrity

The database will be saved and preserved on a secured network at the Concordia University institution. Any inconsistencies in the data will be stated, investigated and resolved. Study staff will be the only individuals with access to the password-protected database. Investigators will permit verification of the data from the ethics board, and maintain sufficient and accurate records of documents. Any changes to the protocol will be reported and communicated to the REB. Confidentiality of the data will be protected and preserved during and after the study. Data will be kept at Concordia University for a minimum of 5 years after the end of the study.

### Sample size justification

While the effect aquatic therapy on paraspinal and gluteal muscles morphology and composition has never been investigated in chronic LBP, previous studies [35, 75, 76], showed significant improvement in multifidus muscle cross-sectional area (CSA) following other exercise interventions with medium to large effect sizes. Based on these studies, we used a mean effect size of 0.73 to calculate our sample size at a level of confidence of 0.05 and 80% power. Accordingly, a sample size of 17 participants in each group will be recruited.

### Statistical analysis

Primary and secondary outcome measures will first be analyzed using descriptive statistics. Changes in paraspinal and gluteal muscle measurements of interest will be assessed for all time points using between-subjects repeated measure analysis of variance (ANOVA). Linear regression models will be used to evaluate the association between paraspinal muscle morphological and functional changes with variations in disability/function status, pain, pain related fear, depression, and anxiety, while accounting for potential confounding factors. Feasibility outcomes related to the use of the “play the pain” application will include adherence rate, and satisfaction will be collected through administration data and end-of-study survey. Survey questions will be answered via a combination of Likert scale, visual analogue scale and will also include questions about barriers and facilitators to the use of the application. Our threshold for adherence and satisfaction outcomes will be 70% out of 100%. Descriptive statistics will be used to summarize the feasibility outcomes.

### Dissemination of findings

The results of this trial will be published in peer-reviewed journals and presented at notable scientific conferences. After publication of manuscripts, data request can be presented to the principal investigator (MF).

## Discussion

LBP is one of the most disabling diseases and carries a large burden globally. Exercise therapy is the most widely used form of treatment for chronic LBP and is recommended as a first-line treatment for the improvement of pain, quality of life, depression and disability/functional status. Aquatic therapy is a more comfortable alternative exercise for people with chronic LBP, with promising results in pain and function. If specific targeted lumbar muscle exercises are to be prescribed and used clinically, the evaluation of physiological muscle changes, such as hypertrophy and reversal of fatty infiltration and whether they mediate improvements in functional status should be considered when assessing the effectiveness of different exercise interventions, such as aquatic therapy and land-based exercise. This study aims to explore the potential benefits of structured water-based exercises that target trunk stabilization and hip muscles on paraspinal and gluteal muscles versus a land-based standard care exercise program. Our research protocol through measurement of muscle morphology and function, clinical symptoms and psychological factors will shed some light into this field.

The results of this trial could have significant implications for clinical practice, enhancing the management and treatment of LBP through the implementation of targeted, cost-effective interventions and clarify whether the use of a digital application in a clinical setting is feasible and can enhance the communication between patients and therapists. This study will generate valuable scientific evidence to support the implementation of a comprehensive LBP program. Such a program could help treat and train individuals with LBP to regain their physical function, mental health, and overall quality of life. Overall, the results of this trial are expected to improve the efficacy of prescriptive exercise training in participants with non-specific chronic LBP.

The limitations of this study include restricting inclusion to those able to understand and read English or French, which will decrease the generalizability of this study. Furthermore, true blinding of exercise supervisors/providers is not possible within an exercise trial.

### Supplementary Information

**Additional file 1:** **Table S1.** Summary of primary and secondary outcomes with follow-up assessments.

## Data Availability

Not applicable.

## Abbreviations

LBP: Low back pain
RCT: Randomized controlled trial
AT: Athletic therapist
MRI: Magnetic resonance imaging
ROI: Region of interest
CSA: Cross-sectional area
FSF: Fat-signal-fraction
ROM: Range of motion
MVIC: Maximum voluntary isometric contraction
SF-12: Short-Form 12 Item survey questionnaire
ODI: Modified Oswestry low back pain disability index
NPRS: The visual numerical Pain Rating Scale
PCS: The Pain Catastrophizing Scale
TSK: The tampa scale of Kinesiophobia
HADS: The hospital anxiety and depression scale
IPAQ: The international physical activity questionnaire
ISI: Insomnia severity index
ANOVA: Analysis of variance
