# Supplementary material for: Effect of aquatic exercise versus standard care on paraspinal and gluteal muscles morphology in individuals with chronic low back pain: a randomized controlled trial protocol
Source: BMC Musculoskelet Disord. 2023 Dec 18;24:977. doi: 10.1186/s12891-023-07034-0 (PMC10726523; doi:10.1186/s12891-023-07034-0)
Supplement: Supplementary file 1 — Additional file 1: Table S1. Summary of primary and secondary outcomes with follow-up assessments. [file 12891_2023_7034_MOESM1_ESM.docx]

**APPENDIX:**

**Table S1:** Summary of primary and secondary outcomes with follow-up assessments.

| Type of measure | Assessments | Measurement time  (weeks) |
| --- | --- | --- |
|  |  | 0 10 |
| **MRI assessment of paraspinal and gluteus muscles morphology** | | |
| Paraspinal and gluteus muscles size (e.g. cross-sectional area CSA). | Measurement of multifidus, erector spinae, psoas, quadratus lumborum, gluteus maximus and medius muscles summative 3D volume or the right and left sides. | x x |
| Paraspinal and gluteus muscle area of lean muscle mass (e.g. excluding fatty infiltration | Measurement of multifidus, erector spinae, psoas, quadratus lumborum gluteus maximum and medius muscles 3D volume of fatty infiltration using a highly reliable thresholding method. | x x |
| Paraspinal muscle and gluteus muscle percent fatty infiltration (e.g. % Fat-signal fraction) | Measurement of multifidus, erector spinae, psoas, quadratus lumborum, gluteus maximum and medius muscles % fat-signal fraction using the DIXON fat and water images. | x x |
| **Muscle strength assessment** | | |
| Lumbar extensors isokinetic maximum muscle strength | Lumbar extensor muscle strength will be evaluated using the MedX Lumbar Isokinetic Dynamometer, in 7 different positions at 12-degree increments. | x x |
| Gluteus maximus and medius muscle strength | Gluteus maximus and medius muscle strength will be assessed using a hand-held dynamometer | x x |
| **Self-reported questionnaire assessments** | | |
| Canadian Minimum data set | Age, gender, height, weight, ethnicity, body mass index, dominant leg, history of LBP (duration, leg pain, pain intensity), history of previous lumbar surgery, smoking and drinking habits, level of education, work status. | x x |
| SF-12 Item Survey | Questionnaire to assess health status and health-related quality of life. | x x |
| Numeric Pain Scale (NPS) | Pain intensity on a 10-point numeric scale | x x |
| Modified Oswestry Disability Index (ODI) | “Gold standard” Low back pain disability questionnaire to measure a patient’s permanent functional disability. | x x |
| Pain Catastrophizing Scale (PCS) | Questionnaire to assess the state of mind of patients with pain. Comprehensive evaluation that encompasses different aspect of catastrophizing. | x x |
| Tampa Scale of Kinesiophobia (TSK) | 17-item questionnaire using a 4-point Likert scale to assess fear of movement, fear of (re)injury, fear avoidance and beliefs about pain in people with chronic pain. | x x |
| Hospital Anxiety and Depression Scale (HADS) | 14-item instrument used to measure the severity of depression and anxiety. | x x |
| International Physical Activity Questionnaire (IPAQ) | Self-reported log of physical activity in minutes per week over a span of 7 days. The level of physical activity is rated as vigorous, moderate, walking and sitting/rest and is assigned to the right category. | x x |
| Insomnia Severity Index (ISI) | 7-item self-reported questionnaire to assess sleep quality. Each question is a Likert-scale from 0 to 4, with lower rating indicating higher quality of sleep. | x x |
